# The global state of research in nonsurgical treatment of knee osteoarthritis: a bibliometric and visualized study

**DOI:** 10.1186/s12891-019-2804-9

**Published:** 2019-09-04

**Authors:** Kai Wang, Dan Xing, Shengjie Dong, Jianhao Lin

**Affiliations:** 1 Arthritis Clinic & Research Center, Peking University People’s Hospital, Peking University, No.11 Xizhimen South Street, Beijing, 100044 China; 2 Arthritis Institute, Peking University, Beijing, China; 3 Orthopedic Department, Yantaishan Hospital, Yantai, Shandong China

**Keywords:** Osteoarthritis, Nonsurgical, Treatment, Bibliometrics, Visualized study

## Abstract

**Background:**

Osteoarthritis (OA) is the most common joint disorder among elderly individuals. Nonsurgical treatment plays an important role in treating knee OA. The aim of the present study was to investigate the trends and research status about nonsurgical treatment of knee OA.

**Methods:**

Publications about the nonsurgical treatment of knee OA from 1994 to 2018 were searched from the Web of Science (WoS) database. The data were analyzed by using bibliometric methodology. The software VOSviewer was used for bibliographic coupling, coauthorship, cocitation, co-occurrence analysis and to investigate the publication trends in nonsurgical treatment of knee OA.

**Results:**

In total, 8512 articles were included. The number of publications increased annually worldwide. The United States has made the largest contribution to this field, with the most publications, citations and the highest H-index. The most contributive institutions were Harvard University, the University of California system and Assistance Publique Hopitaux Paris (APHP). The journal *Osteoarthritis and Cartilage* published the most relative articles. Studies could be classified into five clusters: articular cartilage study, biomechanics study, physiotherapy study, oral pharmacologic study and intra-articular injection study. Articular cartilage and physiotherapy were predicted as the next hot topics in this field.

**Conclusions:**

There will be an increasing number of publications on the nonsurgical treatment of knee OA based on current global trends. The United States made the largest contribution to this field. More focus will be placed on cartilage-related and physiotherapy research, which may be the next popular topics in the nonsurgical treatment of knee OA.

## Background

Osteoarthritis (OA) is the most common joint disorder worldwide, which affects approximately 10% of men and 18% of women over 60 years old [1]. In developed countries, the resultant socioeconomic burden can be 1.0 to 2.5% of gross domestic product [2]. Knee OA is a major source of pain, disability and socioeconomic cost in elderly people. Knee OA pain is a key symptom of persons seeking medical care [3, 4].

The main purpose of knee OA treatment is to alleviate joint pain, improve daily function and quality of life [5]. Although surgical treatment, such as joint replacement, arthroscopic surgery and osteotomy, can be used as an effective procedure for patients with knee OA [6–8], the risk of surgical treatments should not be ignored [9, 10]. Numerous nonsurgical treatments for knee OA are currently available, such as analgesic medicine, physical therapy, wedged insole, unloaded brace and intraarticular injection, which play an important role in relieving joint pain and delaying surgical intervention [11–14]. Intervention for cartilage healing, which included cell-based (with or without scaffolds) or whole-tissue transplantation techniques, may enable us to repair articular cartilage [15–17]. However, the global research trend in the nonsurgical treatment of knee OA has not yet been well studied. Therefore, there is a need to investigate the global status of nonsurgical treatment research in knee OA.

Publications are important indicators of the research trends and contributions of a country or institute. Bibliometrics is a feasible method to evaluate research trends over time qualitatively and quantitatively, based upon the literature databases and literature metrology characteristics. This analysis can help us to grasp the development in a certain research field and evaluate the contribution of journals, institutes and countries [18]. Bibliometric study can also provide evidence for policy and clinical guideline making [19]. The aim of this study was to provide an all-round insight into the current status and global trends of nonsurgical treatment research in knee OA.

## Methods

### Data source and search strategy

Bibliometric analysis was conducted using the Web of Science (WoS) and Science Citation Index-Expanded (SCIE), which are considered as the optimal databases for bibliometrics and cover over 12,000 of the highest impact, quality scientific international journals, providing comprehensive data of publications [20]. All publications were searched in the WoS from 1994 to 2018, which included the articles in this field over the past 25 years. To search for studies on the nonsurgical treatment of knee OA, the most common terms related to surgical treatment of knee OA were excluded. The search strategy was as follows: (theme = osteoarthritis AND theme = knee Not theme = arthroplasty Not theme = replacement Not theme = unicompartment Not theme = arthroscopic Not theme = osteotomy) AND (theme = treatment OR theme = therapy). All publications identified were English language.

### Selection criteria and data collection

The inclusion criteria were articles related to the nonsurgical treatment of knee OA (such as the mechanism, outcome, side effect and patients’ adherence etc.), covering all types of studies. The exclusion criteria were the articles that were not relevant to nonsurgical treatment or knee OA (i.e false-positive data). We also excluded trials published only as abstracts (with no additional data available from other sources).

The information of all eligible publications including title, author, year of publication, nationalities, affiliations, journal, keywords and abstract, was downloaded from the WoS database. Two authors (Wang K and Xing D) independently verified the data collection and entry. The disagreement was resolved by the third author (Lin JH) to reach a consensus.

### Bibliometric analysis

The basic characteristics of publications were described by using the intrinsic function of WoS. The H-index, a useful measure of the impact of scientific study, shows that a scientist or scholar has published H papers, each of which has been cited at least H times by other publications. Thus, the H-index reflects both the number and citation impact of publications [21, 22]. Relative research interest (RRI) is defined as the number of publications in a certain field divided by all-field publications per year. The time trend of the publications was analyzed by using R software (version 3.1.3). The logistic regression model: *f(x)* = *c*/[*1* + *a* × *exp*(−*b* × (*x-1994)*)] was used to model the cumulative volume of documentation. In this formula, the symbol *x* refers to the year, and *f(x)* represents the cumulative amount of papers. The time when the growth rate of publications changed from positive to negative was defined as the inflection point, which was generated using the formula *T* = *lna/b + 1994.*

### Visualized analysis

VOSviewer software (Leiden University, Leiden, The Netherlands) was used for the bibliometric visualization and analysis of the literature [23]. In this research, VOSviewer was used for cocitation, coauthorship, bibliographic coupling and co-occurrence analysis.

## Results

### Global publication trends

#### Number of global publications

**Fig. 1:**
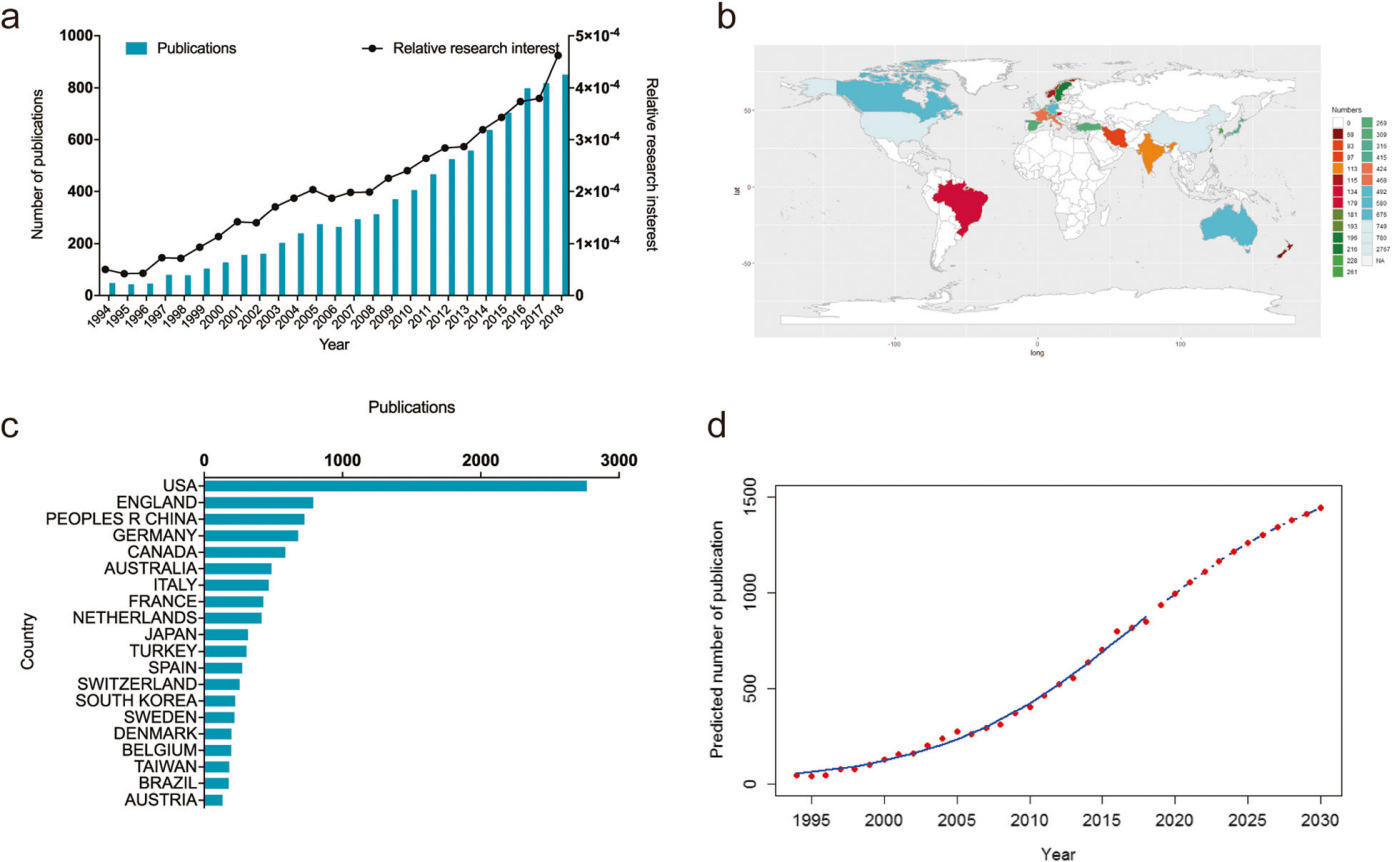
**a** The total number and RRIs of publications related to nonsurgical treatment of knee OA study. The blue bars mean the number of publications each year, and the black curve means the RRIs. **b** Distribution of nonsurgical treatment of knee OA research in world map. **c** The top 20 countries of total number of publications. **d** Model fitting curves of trends in global publications

In total, 8512 publications met the criteria. From 1994 to 2018, there has been a steadily increasing trend of global publications annually. The number of publications increased from 46 (1994) to 849 (2018). Most research was published in 2018 (849, 9.97%) (Fig. 1a).

#### Contributions of countries and regions

In total, 78 countries and regions published articles in this domain. The United States published the most papers (2766, 32.50%), followed by England (788, 9.26%), China (725, 8.52%) and Germany (679, 7.98%). (Fig. 1b and c).

#### Global trends of publications

The time curve of the number of the publications was created by using the logistic regression model, which could help predict the future trend. Figure 1d showed the model fitting curves of the growth trend. On the basis of the time curve, the number of publications in this field was estimated to grow by nearly 30 times from 46 in 1994 to approximately 1500 by 2030.

### Quality of the publications

**Fig. 2:**
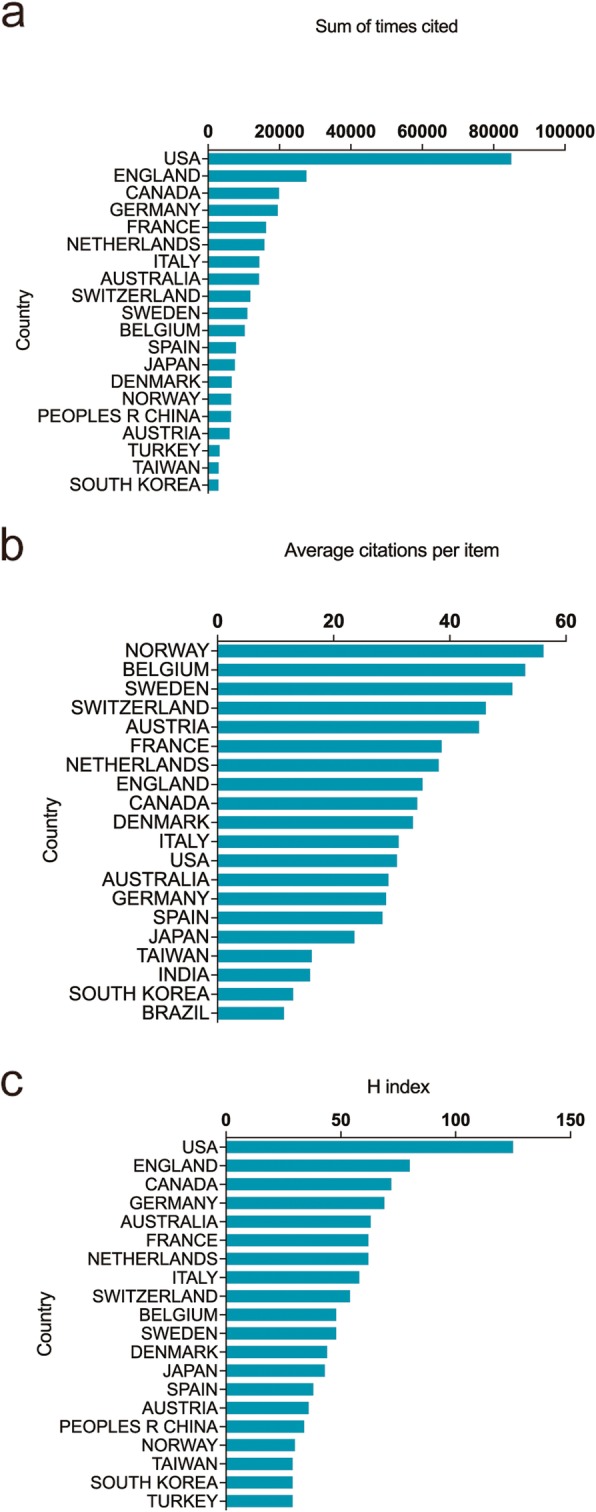
**a** The top 20 countries of total citations. **b** The top 20 countries of the average citations for each article. **c** The top 20 countries of the H-index of publications

Papers from the United States had the highest citation frequencies (84,952). England ranked second in total citation frequency (27,536), followed by Canada (19,871), Germany (19,516) and France (16,248) (Fig. 2a). The average citation frequencies of top 20 countries were shown in Fig. 2b. Norway had the highest average citation frequencies (56.15), followed by Belgium (52.97) and Sweden (50.77).

As far as the H-index was concerned, United States outranked other countries with H-index of 125, followed by England (80), Canada (72), Germany (69) and Australia (63) (Fig. 2c).

### Analysis of global publication

#### Journal analysis

**Fig. 3:**
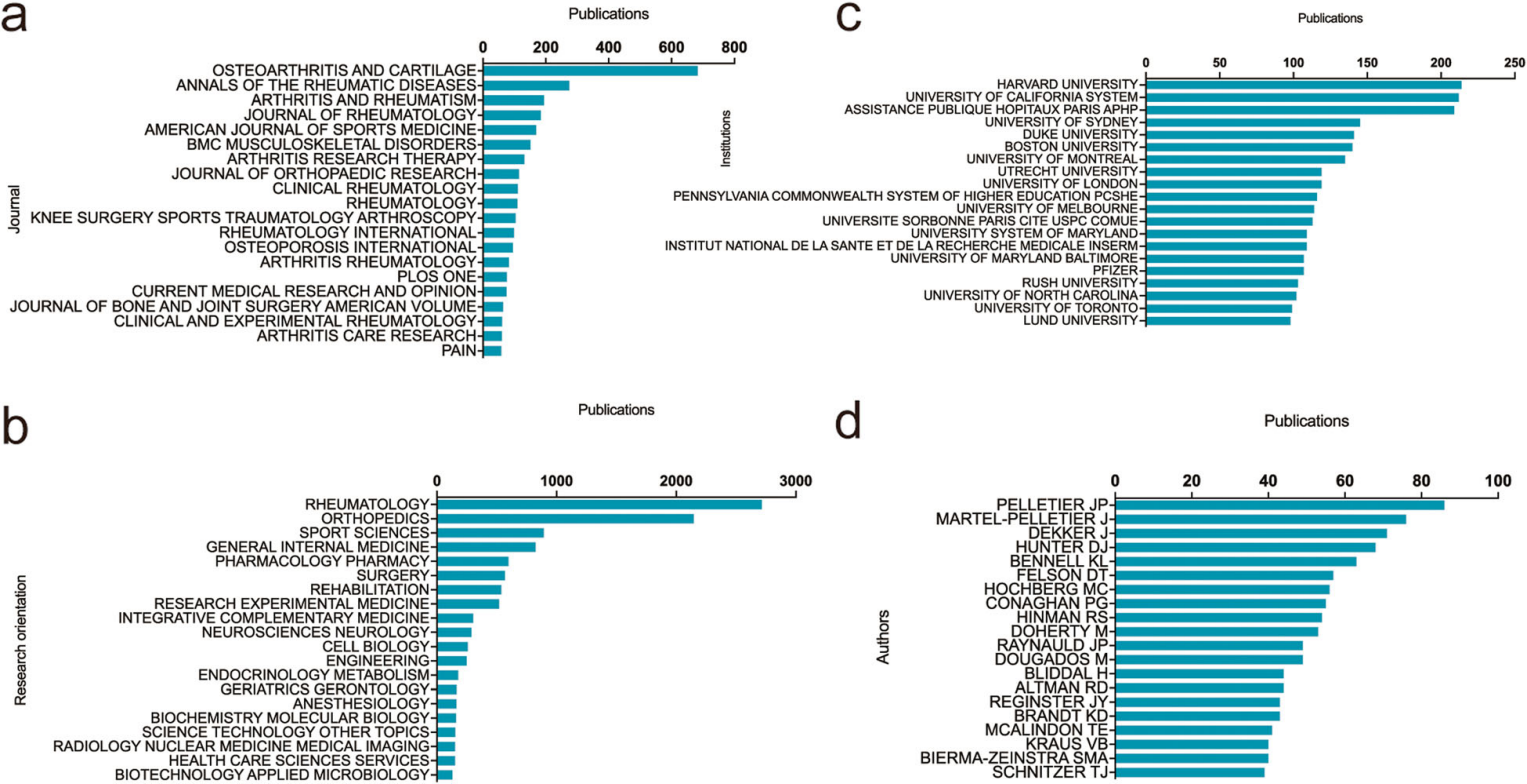
**a** The top 20 journals with most publications related to the nonsurgical treatment of knee OA. **b** The 20 main research orientations and the number of publications in each orientation. **c** The top 20 institutions of high-impact and the number of their publications. **d** The top 20 authors with most publications in this field

OSTEOARTHRITIS AND CARTILAGE published the most articles, with 683 publications. There were 275 articles in ANNALS OF THE RHEUMATIC DISEASES, 194 articles in ARTHRITIS AND RHEUMATISM and 184 articles in THE JOURNAL OF RHEUMATOLOGY on the nonsurgical treatment of knee OA. The top 20 journals with most papers were shown in Fig. 3a.

#### Research orientation

Figure 3b showed the top 20 research orientations related to nonsurgical treatment of knee OA. The most prevalent areas of research were rheumatology, orthopedics, sports science and general internal medicine.

#### Institution output

The institution with the greatest number of publications was Harvard University with 214 papers. The University of California system ranked second (212 papers), followed by the Assistance Publique Hopitaux Paris (APHP) (209 papers). The top 20 institutions with the most articles were shown in Fig. 3c.

#### Authors

The top 20 authors published a total of 1071 papers, which accounted for 12.58% of all literature in this field (Fig. 3d). Pelletier JP published the most research on OA and nonsurgical treatment with 86 papers, followed by Martel-Pelletier J with 76 papers and Dekker J with 71 papers. In this study, we included all authors for the analysis and did not consider the authors’ relative contribution to one research.

### Bibliographic coupling analysis

#### Journals

**Fig. 4:**
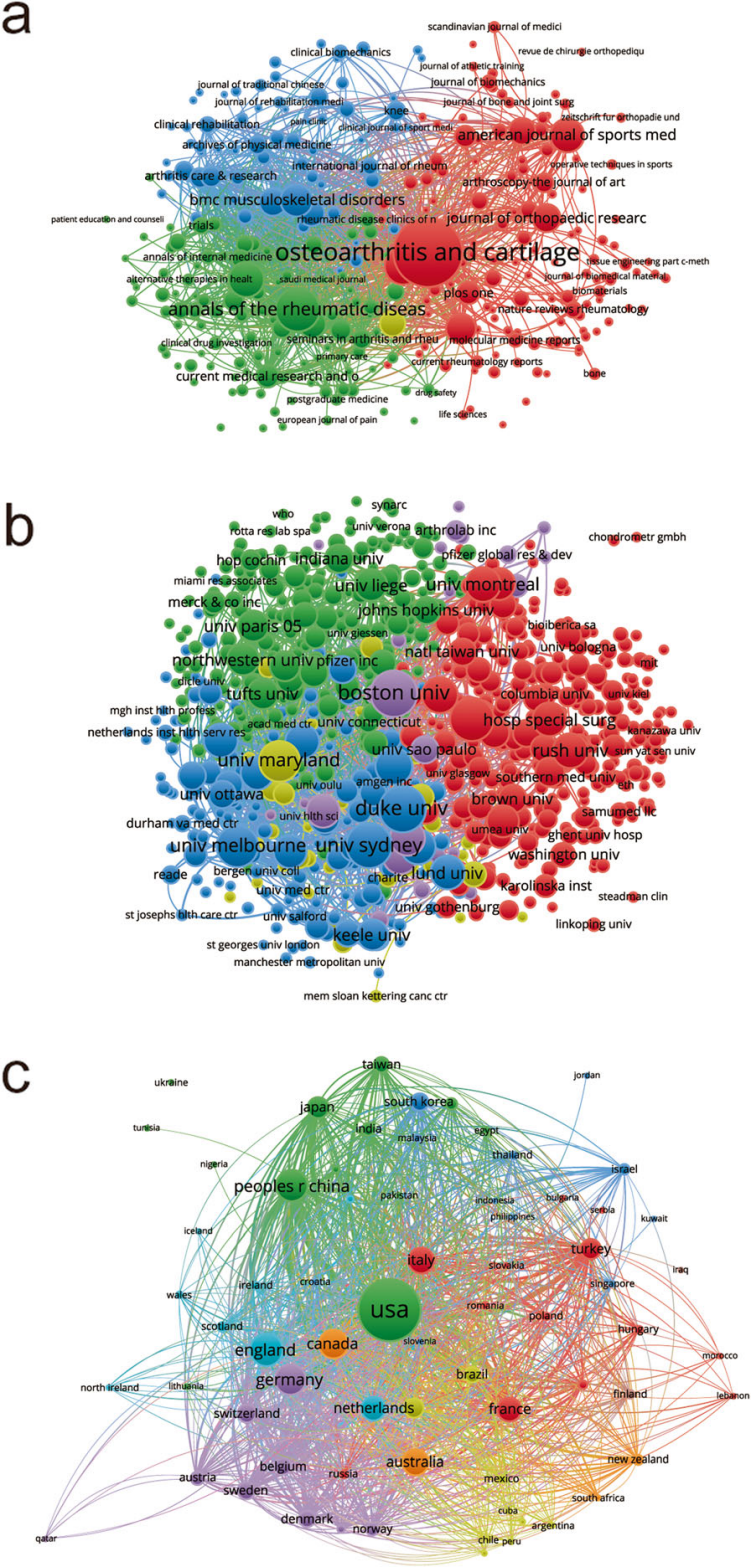
Bibliographic coupling analysis of worldwide research on nonsurgical treatment of knee OA. **a** Mapping on the 315 identified journals on nonsurgical treatment of knee OA. **b** Mapping on 815 institutions on nonsurgical treatment of knee OA. **c** Mapping on the 69 countries in this field. The line between two icons in the figure indicates that two journals/institutions/countries had created a similarity relationship

Bibliographic coupling analysis is a well-established method which helps create a relationship of document similarity based on the number of references they share. The VOSviewer was used to analyze the names of journals in total publications. As illustrated in Fig. 4a, 315 journals appeared in the bibliometric map. The top five journals with greatest total link strengths were as follows: OSTEOARTHRITIS AND CARTILAGE (525,463 times), BMC MUSCULOSKELETAL DISORDERS (228,254 times), ANNALS OF THE RHEUMATIC DISEASES (212,472 times), THE JOURNAL OF RHEUMATOLOGY (198,529 times) and CLINICAL RHEUMATOLOGY (140,629 times).

#### Institutions

There were 815 institutions included (the minimum number of publications of an institution was over five) and their publications were analyzed by VOSviewer (Fig. 4b). The top five institutions with greatest total link strengths were as follows: University of Sydney (488,866 times), University of Melbourne (486,697 times), Boston University (390,756 times), Duke University (331,637 times), and University of Maryland (279,289 times).

#### Countries

There were 69 countries included (the minimum number of publications from a country was over five) and their publications were analyzed by VOSviewer (Fig. 4c). The top five countries with greatest total link strengths were as follows: the United States (3,298,992 times), England (1,230,270 times), Australia (1,074,537 times), Canada (1,003,319 times), and Germany (927,904 times).

### Coauthorship analysis

#### Authors

**Fig. 5:**
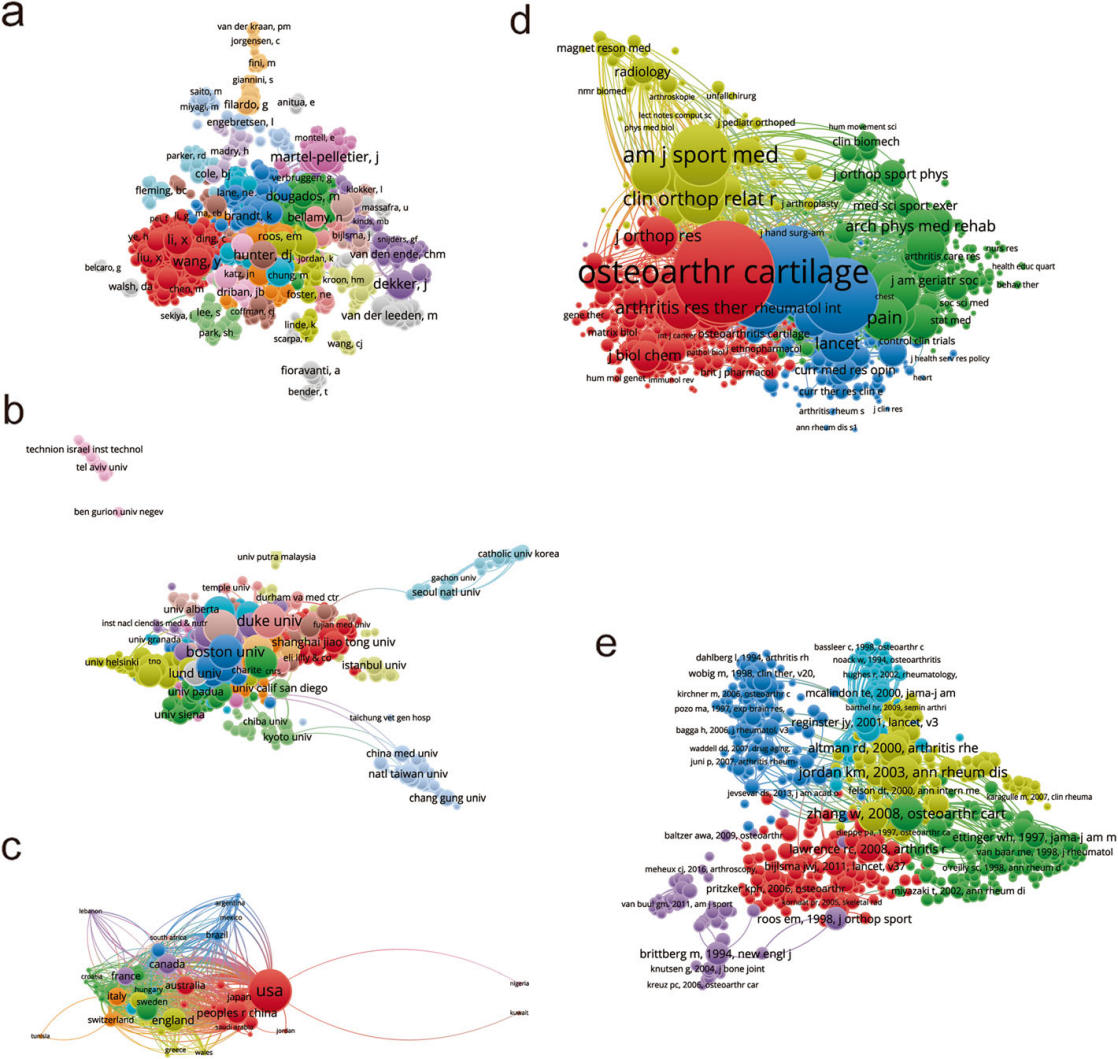
Coauthorship and cocitation analysis of global research about nonsurgical treatment of knee OA. **a** Mapping on the 1447 identified authors by coauthorship analysis on nonsurgical treatment of knee OA. **b** Mapping on the 816 identified institutions by coauthorship analysis on nonsurgical treatment of knee OA. **c** Mapping on the 69 identified countries by coauthorship analysis on nonsurgical treatment of knee OA. The size of the icons indicates the coauthorship frequency. The line between two icons in the figure indicates that two authors/institutions/countries had established collaboration. **d** Mapping on the 1470 identified journals by cocitation analysis. The size of the icons indicates the citation frequency. A line between two icons indicates that both were cited in one journal. **e** Mapping on the 1730 identified references by cocitation analysis. The size of the icons indicates the citation frequency. A line between two icons indicates that both were cited in one paper

Coauthorship analysis means that the relationship of items is built according to the number of coauthored documents; 1447 authors were identified (the minimum number of publications from an author was over five) and their publications were analyzed by VOSviewer (Fig. 5a). The top five authors with greatest total link strengths were as follows: Martel-Pelletier J (361 times), Dekker J (344 times), Pelletier JP (334 times), Li X (289 times), and Wang Y (246 times).

#### Institutions

The publications in 816 identified institutions (the minimum number of publications from an institution was over five) were analyzed by VOSviewer (Fig. 5b). The top five institutions with greatest total link strengths were as follows: Boston University (340 times), University of Sydney (331 times), University of Maryland (276 times), University of Oxford (264 times) and Duke University (263 times).

#### Countries

The studies in 69 identified countries (the minimum number of studies from a country was over five) were analyzed by VOSviewer (Fig. 5c). The top five countries with greatest total link strengths were as follows: the United States (1456 times), England (897 times), Germany (597 times), Canada (570 times) and France (530 times).

### Cocitation analysis

#### Journals

Cocitation analysis means that the relationship of items is built based upon the number of times they are cited together. VOSviewer was used to perform the cocitation analysis of the journals. The journal was included if the minimum number of citations from a source was over 20 times. There were 1470 journals that met the criteria (Fig. 5d). The top five journals with greatest total link strengths were as follows: OSTEOARTHRITIS AND CARTILAGE (970,864 times), ANNALS OF THE RHEUMATIC DISEASES (641,747 times), ARTHRITIS AND RHEUMATOLOGY (597,111 times), THE JOURNAL OF RHEUMATOLOGY (497,631 times) and AMERICAN JOURNAL OF SPORTS MEDICINE (314,811 times).

#### Publications

A total of 1730 publications (the minimum number of citations of a reference was over 20 times) were analyzed by using VOSviewer (Fig. 5e). The top five publications with greatest total link strengths were as follows: Jordan et al. [24] (7945 times); Zhang et al. [25] (7821 times); Hochberg et al. [26] (5721 times); Zhang et al. [27] (4845 times) and McaAlindon et al. [28] (4589 times).

### Co-occurrence analysis

**Fig. 6:**
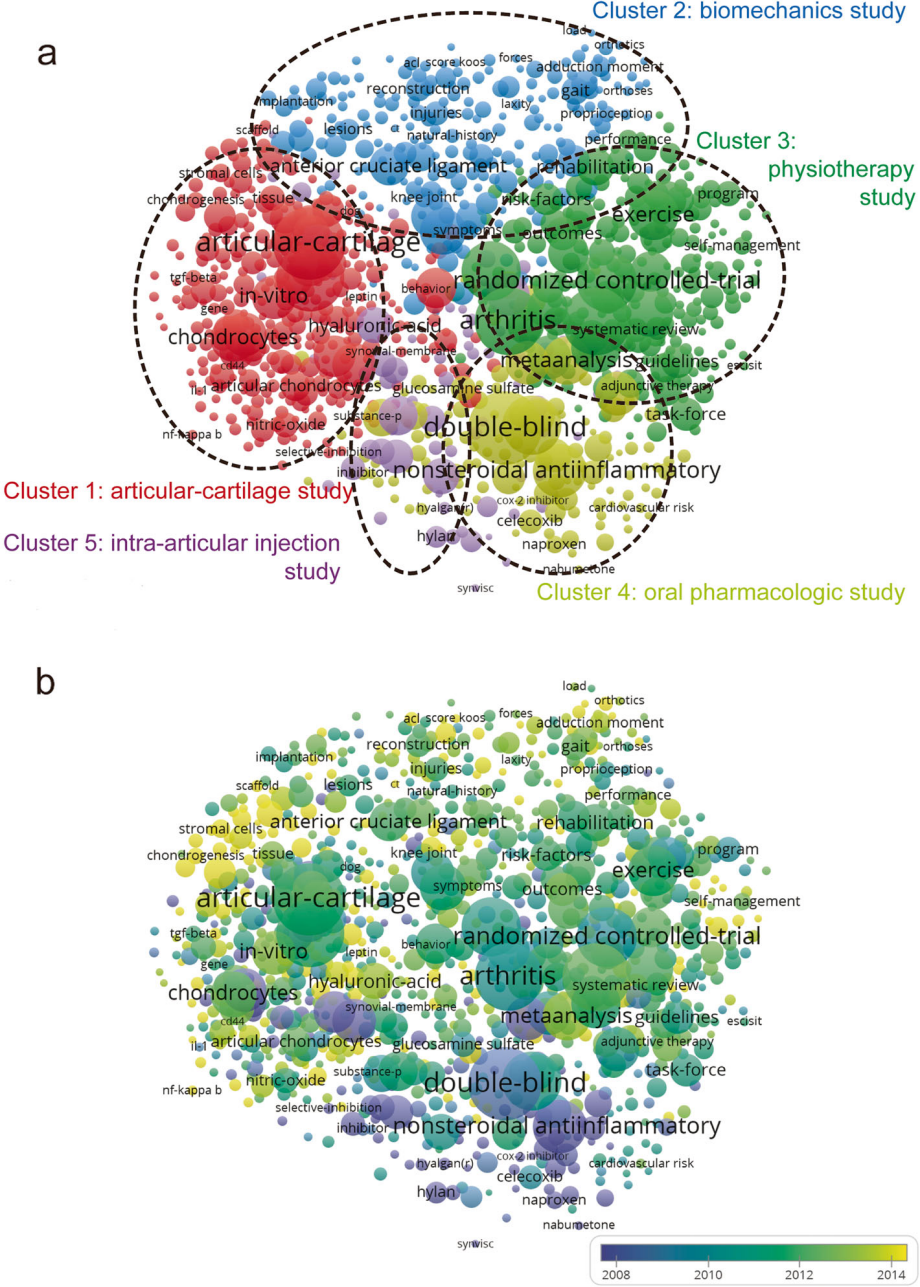
Co-occurrence analysis of nonsurgical treatment of knee OA. **a** Mapping on keywords in the study on nonsurgical treatment of knee OA; the size of the icons indicates the frequency, and the keywords are classified into five clusters: articular cartilage study (red color), biomechanics study (blue color), physiotherapy study (green color), oral pharmacologic study (yellow color) and intra-articular injection study (purple color). **b** Distribution of keywords according to their time of appearance; keywords in purple appeared earlier than those in green, and keywords in yellow appeared later

Co-occurrence analysis indicates that the relationship of items is built based upon the number of publications in which they occur together. The goal of this analysis is to determine research areas and hot issues, and it is an important indicator to track the scientific development [29]. The keywords (the minimum number of occurrences of a keyword was over five) were analyzed by VOSviewer. As illustrated in Fig. 6a, the 980 identified keywords could be divided into five clusters: “articular cartilage study”, “biomechanics study”, “physiotherapy study”, “oral pharmacologic study” and “intra-articular injection study” (Fig. 6a). In the “articular cartilage study” cluster, the most used keywords were articular cartilage, chondrocytes, mesenchymal stem cells and gene expression. In the cluster of “biomechanics study”, the primary keywords were anterior cruciate ligament, gait, progression and proprioception. In the “physiotherapy study” cluster, the main keywords were management, exercise, rehabilitation and recommendations. In the “oral pharmacologic study” cluster, the frequently used keywords were nonsteroidal anti-inflammatory, double-blinded, placebo and efficacy. In the cluster of “intra-articular injection study”, the main keywords were injection, hyaluronic acid, sodium hyaluronate and viscosupplementation. These results showed the research field distribution of publications related to nonsurgical treatment of knee OA.

In Fig. 6b, VOSviewer applied different colors to keywords based on when they appeared in literature for the average time. The purple color indicated that the keywords appeared earlier while keywords in yellow color appeared later. Before 2010, in the early stage of this research field, most studies focused on “oral pharmacologic study” and “intra-articular injection study”. Recent trends indicated that two categories of research, “physiotherapy study” and “articular cartilage study”, might be studied more in future.

## Discussion

### Global trends in nonsurgical treatment of knee OA research

Bibliometrics and visualized research can be used to show the current status and predict the future development in research. Therefore, the purpose of this study was to investigate nonsurgical treatment studies in knee OA with respect to global trends of publications, contributing countries, institutions and research aims. As shown in this study, there have been a significant increasing number of publications over years. In addition, the RRIs have increased dramatically over recent years. According to the current data, more studies on the nonsurgical treatment of knee OA will be published in the next few years.

### Status and quality of global publications

The H-index and total number of citations represent the academic impact and quality of publications of a country [30]. The United States was the leading country in both the total number of publications and total citation frequency. England also made a tremendous contribution to this field when considering the total number of papers and the total citation frequency. Some countries——such as Norway and Belgium——have also played an important role because of their high average citation frequencies.

The OSTEOARTHRITIS AND CARTILAGE, ANNALS OF THE RHEUMATIC DISEASES, ARTHRITIS AND RHEUMATISM and THE JOURNAL OF RHEUMATOLOGY published the most studies on the nonsurgical treatment of knee OA. Future findings in this field are more likely to be reported by the listed journals (Fig. 4a).

Almost all of the top 20 institutions were from the top five countries, implying that establishing first-class research institutions is essential to improving a country’s academic level. Pelletier JP, Martel-Pelletier J and Dekker J were the top three authors who published the most articles in this field. The top 20 authors could be regarded as the pioneers in the nonsurgical treatment of knee OA. Their future studies may have a substantial impact on the development in this field and should be closely monitored to grasp the latest advancement.

In this study, the relatedness between papers with regards to country, institution and journal was established by bibliographic coupling analysis. Bibliographic coupling was constructed on the shared references among publications, providing deeper insights on how authors use and build links among the existing literature. The analysis showed that OSTEOARTHRITIS AND CARTILAGE published the most relevant articles, and the United States was in the leading position in this field. Coauthorship analysis was used to assess collaboration among countries, institutions and authors. The one with greater total link strength suggested that the country/institution/author would be more likely to cooperate with others. Cocitation analysis was conducted to evaluate the academic influence of studies. The top studies with larger total cocitation frequency could be regarded as the landmark studies about the nonsurgical treatment of knee OA. OSTEOARTHRITIS AND CARTILAGE was the journal with the highest citation frequency in this domain.

### Research focus on the nonsurgical treatment of knee OA

The research directions and hotspots in this field were identified by using the co-occurrence analysis. The map of the co-occurrence network was created based on the keywords of all included studies. As shown in Fig. 6a, five research directions were discovered, including articular cartilage study, biomechanics study, physiotherapy study, oral pharmacologic study and intra-articular injection study. This result could help clarify the trend of future research. In the co-occurrence map, the keywords, including arthritis, articular cartilage, exercise and management, were highlighted with bigger icons. Thus, further high-quality studies concerning articular cartilage study and physiotherapy study may still be required.

The overlay visualization map was color coded by VOSviewer based on the average time the keywords appeared in the publications [31]. This was an important method for monitoring the progress of research. In this overlay visualization (Fig. 6b), the color bar indicated the year of publication. According to the results, articular cartilage and physiotherapy (yellow color) may be the next hot topic in this field. Studies on articular cartilage, especially the development of regenerative medicine and tissue engineering techniques to restore articular cartilage, have been widely emerged [15, 32–34]. Additional research is required to develop truly biomimetic cartilage regenerative therapies and translate these approaches to clinical practice [35, 36]. Physiotherapy, like exercise, is recommended by many guidelines for osteoarthritis [26, 28, 37], which plays a key role in preventing osteoarthritis and improving the daily function and quality of life of patients. Primary studies investigating the efficacy and individualization of physiotherapy for knee OA remain the focus of this research field.

Based on the results of our study, the increasing number of publications indicated that the nonsurgical treatment serves a crucial position in managing knee OA. This promising result will also in turn encourage investigators to conduct further research. The bibliometric and visualized analyses could provide the researchers with the knowledge of the leading countries, authors and institutions in this field. With these information, the researchers could have the ‘main channel’ to get the advanced knowledge and future discoveries. Additionally, the co-occurrence analysis and overlay visualization map specifically delineated the hotspots and future research directions, which may help the funding agencies to make a more rational investment plan and provide the evidence for health-care policy making.

### Strengths and limitations

By using bibliometric and visualized analyses, the present study gave a deep insight into the worldwide status and trends of studies about the nonsurgical treatment of knee OA. However, the following limitations must be mentioned. The database variation is the limitation of bibliometric analysis. It is widely known that the publications from major databases such as WoS, Pubmed, Embase and Cochrane library differ. Therefore, we may omit some publications due to the database bias. Besides, We only included English language studies based on WoS. Non-English publications, such as Chinese, have been excluded. Considering the large population of OA patients in these countries, excluding the non-English publications may lead to language bias. In addition, there may exist differences between the bibliometric analysis results and real-world research conditions. For instance, some recently published high-quality papers might not be emphasized due to low citation frequency. Therefore, there is still a need to pay attention to the latest published papers and other non-English publications in the daily research.

## Conclusions

This study showed the current status and global trends in the nonsurgical treatment of knee OA. The United States was the leading country in both the total number of publications and total citation frequency. The journal OSTEOARTHRITIS AND CARTILAGE published the most papers related to this issue. It can be predicted that there will be an increasing number of papers in the coming year. In particular, studies about articular cartilage and physiotherapy will be the next popular hotspots and receive more attention in the future.

## Data Availability

Data will be available upon request by the first author KW.

## Abbreviations

APHP: Assistance Publique Hopitaux Paris
OA: Osteoarthritis
RRI: Relative research interest
SCIE: Science Citation Index-Expanded
WoS: Web of Science

## References

[1] Mandl L.A. (2019). Osteoarthritis year in review 2018: clinical. Osteoarthritis and Cartilage.

[2] Silverwood V, Blagojevic-Bucknall M, Jinks C (2015). Current evidence on risk factors for knee osteoarthritis in older adults: a systematic review and meta-analysis. Osteoarthr Cartil.

[3] Glyn-Jones S, Palmer AJ, Agricola R (2015). Osteoarthritis Lancet.

[4] Flemming DJ, Gustas-French CN (2017). Rapidly progressive osteoarthritis: a review of the clinical and radiologic presentation. Curr Rheumatol Rep.

[5] Roos EM, Arden NK (2016). Strategies for the prevention of knee osteoarthritis. Nat Rev Rheumatol.

[6] Buchbinder R, Richards B, Harris I (2014). Knee osteoarthritis and role for surgical intervention: lessons learned from randomized clinical trials and population-based cohorts. Curr Opin Rheumatol.

[7] Lohkamp M, Schmitt H, Kromer TO (2017). Osteoarthritis and joint replacements of the lower limb and spine in ex-professional soccer players: a systematic review. Scand J Med Sci Sports.

[8] Hussain SM, Neilly DW, Baliga S (2016). Knee osteoarthritis: a review of management options. Scott Med J.

[9] Passias PG, Bono OJ, Bono JV. Total knee arthroplasty in patients of advanced age: a look at outcomes and complications. J Knee Surg. 2018. 10.1055/s-0038-1676067. 10.1055/s-0038-1676067 30477044

[10] Abram SGF, Judge A, Beard DJ (2018). Adverse outcomes after arthroscopic partial meniscectomy: a study of 700 000 procedures in the national hospital episode statistics database for England. Lancet..

[11] Crawford DC, Miller LE, Block JE (2013). Conservative management of symptomatic knee osteoarthritis: a flawed strategy?. Orthop Rev (Pavia).

[12] Filardo G, Kon E, Longo UG (2016). Non-surgical treatments for the management of early osteoarthritis. Knee Surg Sports Traumatol Arthrosc.

[13] Kamaruzaman H, Kinghorn P, Oppong R (2017). Cost-effectiveness of surgical interventions for the management of osteoarthritis: a systematic review of the literature. BMC Musculoskelet Disord.

[14] Xing D, Zhao Y, Dong S (2018). Global research trends in stem cells for osteoarthritis: a bibliometric and visualized study. Int J Rheum Dis.

[15] Makris EA, Gomoll AH, Malizos KN (2015). Repair and tissue engineering techniques for articular cartilage. Nat Rev Rheumatol.

[16] de Girolamo L, Kon E, Filardo G (2016). Regenerative approaches for the treatment of early OA. Knee Surg Sports Traumatol Arthrosc.

[17] Li MH, Xiao R, Li JB (2017). Regenerative approaches for cartilage repair in the treatment of osteoarthritis. Osteoarthr Cartil.

[18] Pu QH, Lyu QJ, Su HY (2016). Bibliometric analysis of scientific publications in transplantation journals from mainland China, Japan, South Korea and Taiwan between 2006 and 2015. BMJ Open.

[19] Avcu G, Sahbudak Bal Z, Duyu M (2015). Thanks to trauma: a delayed diagnosis of Pott disease. Pediatr Emerg Care.

[20] Aggarwal A, Lewison G, Idir S (2016). The state of lung Cancer research: a global analysis. J Thorac Oncol.

[21] Bornmann L, Daniel HD (2009). The state of h index research. Is the h index the ideal way to measure research performance?. EMBO Rep.

[22] Bertoli-Barsotti L, Lando T (2017). A theoretical model of the relationship between the h-index and other simple citation indicators. Scientometrics..

[23] Synnestvedt MB, Chen C, Holmes JH (2005). CiteSpace II: visualization and knowledge discovery in bibliographic databases. AMIA Annu Symp Proc.

[24] Jordan KM, Arden NK, Doherty M (2003). EULAR recommendations 2003: an evidence based approach to the management of knee osteoarthritis: report of a task force of the standing committee for international clinical studies including therapeutic trials (ESCISIT). Ann Rheum Dis.

[25] Zhang W, Moskowitz RW, Nuki G (2008). OARSI recommendations for the management of hip and knee osteoarthritis, part II: OARSI evidence-based, expert consensus guidelines. Osteoarthr Cartil.

[26] Hochberg MC, Altman RD, April KT (2012). American College of Rheumatology 2012 recommendations for the use of nonpharmacologic and pharmacologic therapies in osteoarthritis of the hand, hip, and knee. Arthritis Care Res (Hoboken).

[27] Zhang W, Nuki G, Moskowitz RW (2010). OARSI recommendations for the management of hip and knee osteoarthritis: part III: changes in evidence following systematic cumulative update of research published through January 2009. Osteoarthr Cartil.

[28] McAlindon TE, Bannuru RR, Sullivan MC (2014). OARSI guidelines for the non-surgical management of knee osteoarthritis. Osteoarthr Cartil.

[29] Gao Y, Wang Y, Zhai X (2017). Publication trends of research on diabetes mellitus and T cells (1997-2016): a 20-year bibliometric study. PLoS One.

[30] Bastian S, Ippolito JA, Lopez SA (2017). The use of the h-index in academic Orthopaedic surgery. J Bone Joint Surg Am.

[31] Hesketh KR, Law C, Bedford H (2016). Co-occurrence of health conditions during childhood: longitudinal findings from the UK millennium cohort study (MCS). PLoS One.

[32] Lories RJ, Luyten FP (2011). The bone-cartilage unit in osteoarthritis. Nat Rev Rheumatol.

[33] Walter Sebastian G., Ossendorff Robert, Schildberg Frank A. (2018). Articular cartilage regeneration and tissue engineering models: a systematic review. Archives of Orthopaedic and Trauma Surgery.

[34] Mamidi MK, Das AK, Zakaria Z (2016). Mesenchymal stromal cells for cartilage repair in osteoarthritis. Osteoarthr Cartil.

[35] Fisher JN, Tessaro I, Bertocco T (2017). The application of stem cells from different tissues to cartilage repair. Stem Cells Int.

[36] Xing D, Wang Q, Yang Z (2018). Mesenchymal stem cells injections for knee osteoarthritis: a systematic overview. Rheumatol Int.

[37] Brown GA (2013). AAOS clinical practice guideline: treatment of osteoarthritis of the knee: evidence-based guideline, 2nd edition. J Am Acad Orthop Surg.

